# Multifunctional Biomedical Devices with Closed‐Loop Systems for Precision Therapy

**DOI:** 10.1002/adhm.202500860

**Published:** 2025-06-30

**Authors:** Yixuan Leng, Rujie Sun

**Affiliations:** ^1^ Department of Engineering Science Institute of Biomedical Engineering (IBME) University of Oxford Oxford OX3 7DQ UK; ^2^ School of Electronics and Computer Science (ECS) University of Southampton Southampton SO17 1BJ UK

**Keywords:** bioelectronics, biomaterials, closed‐loop, drug delivery, stimuli‐responsive

## Abstract

Closed‐loop control systems have emerged as transformative tools in precision therapy, enabling real‐time monitoring of patient's physiological conditions and automatically adjusting treatments based on direct feedback. By seamlessly integrating sensing feedback and on‐demand therapeutic interventions, these systems offer enhanced accuracy, adaptability, and effectiveness, while reducing the risks of over‐ or under‐treatment. This review categorizes closed‐loop devices into two major types: self‐sustained and externally triggered. It first examines the stimuli‐responsive materials and mechanisms essential for self‐sustained systems, which autonomously deliver therapeutic agents in response to physiological or environmental cues without external intervention, an approach that is particularly advantageous for managing chronic diseases. The discussion then focuses on recent developments in integrated bioelectronics as a platform for externally triggered closed‐loop systems, summarising key innovations in biosensing technologies, personalized therapeutic strategies, and data‐driven control algorithms. Finally, the review outlines current challenges and highlights potential research avenues, illustrating the transformative potential of closed‐loop systems in a range of precision therapies, including diabetes management and neurostimulation.

## Graphical abstract

This review explores two major types of closed‐loop therapeutic systems: self‐sustained and externally triggered. It highlights recent advances in stimuli‐responsive materials, integrated bioelectronics, and data‐driven control strategies, offering insight into emerging strategies for personalized, responsive drug delivery. Key applications in chronic disease management and neurostimulation illustrate the potential of these technologies for precision medicine.

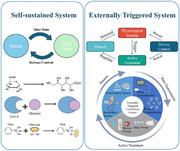

## 1 Introduction

In recent years, advancements in material science and microfabrication have paved the way for the development of biomedical tools, enabling transformative solutions in precision therapy across a wide range of applications, including sensing, diagnosis, and treatment. Among these innovations, closed‐loop control systems have emerged as powerful tools for enhancing treatment precision and effectiveness. For instance, various medical devices now enable real‐time monitoring of biological signals in real‐time, including glucose levels,^[^ ^1^, ^2^, ^3^, ^4^, ^5^ ^]^ heart rate,^[^ ^6^, ^7^, ^8^ ^]^ and neural activity.^[^ ^9^, ^10^ ^]^ These real‐time monitoring capabilities not only allow for early disease detection but are also essential to improve therapeutic outcomes. With closed‐loop controlled systems, treatments such as neurostimulation and drug delivery can provide targeted and controlled interventions, effectively treating conditions like epilepsy,^[^ ^11^, ^12^, ^13^ ^]^ chronic pain,^[^ ^14^, ^15^, ^16^ ^]^ and Parkinson's disease.^[^ ^17^ ^]^ The integration of these technologies holds immense potential to revolutionize healthcare by making treatment more adaptive, personalized, and efficient.

Traditional treatments often rely on open‐loop systems,^[^ ^18^, ^19^, ^20^ ^]^ where interventions, such as drug administration or electrical stimulation are delivered without real‐time feedback from the body. These approaches can result in over‐ or under‐dosing, as they cannot adapt to a patient's changing physiological state.^[^ ^21^ ^]^ For example, a patient with diabetes using a traditional insulin pump may receive insulin at a predetermined rate, regardless of fluctuating blood sugar levels throughout the day, potentially leading to imbalances.^[^ ^22^ ^]^ To achieve better treatment for various diseases, the development of advanced, well‐controlled biomedical tools has become increasingly important. Closed‐loop systems, compared with open‐loop ones, offer a more efficient solution by continuously monitoring specific biomarkers, allowing the system to adjust its output dynamically.^[^ ^22^, ^23^ ^]^ By integrating real‐time physiological signals into the treatment process, these systems maintain optimal therapeutic levels, thereby personalizing care and improving patient outcomes across a range of diseases.

Multifunctional biomedical devices for closed‐loop precision therapy, as an interdisciplinary field, combine biomaterials, electronics, soft robotics, and control systems, leading to significant advancements in healthcare engineering. By continuously monitoring a patient's condition and dynamically adjusting therapeutic interventions, closed‐loop systems maintain physiological processes within a desired range. Several strategies have been developed to achieve closed‐loop precision therapy. For example, stimuli‐responsive systems use materials that react to environmental changes, triggered by internal signals such as pH, glucose, or enzymes. These systems can automatically release treatments when needed, ensuring timely interventions. Additionally, advancements in bioelectronic devices have enabled the integration of various biosensors to collect health data, process it, and deliver targeted treatments tailored to individual patient needs. While challenges such as accurate and reliable sensing remain, these systems exhibit excellent adaptability, enabling precise and personalized drug delivery. This reduces the risks of under or over‐dosing, optimizing therapeutic outcomes and enhancing patient care.

**Figure 1:**
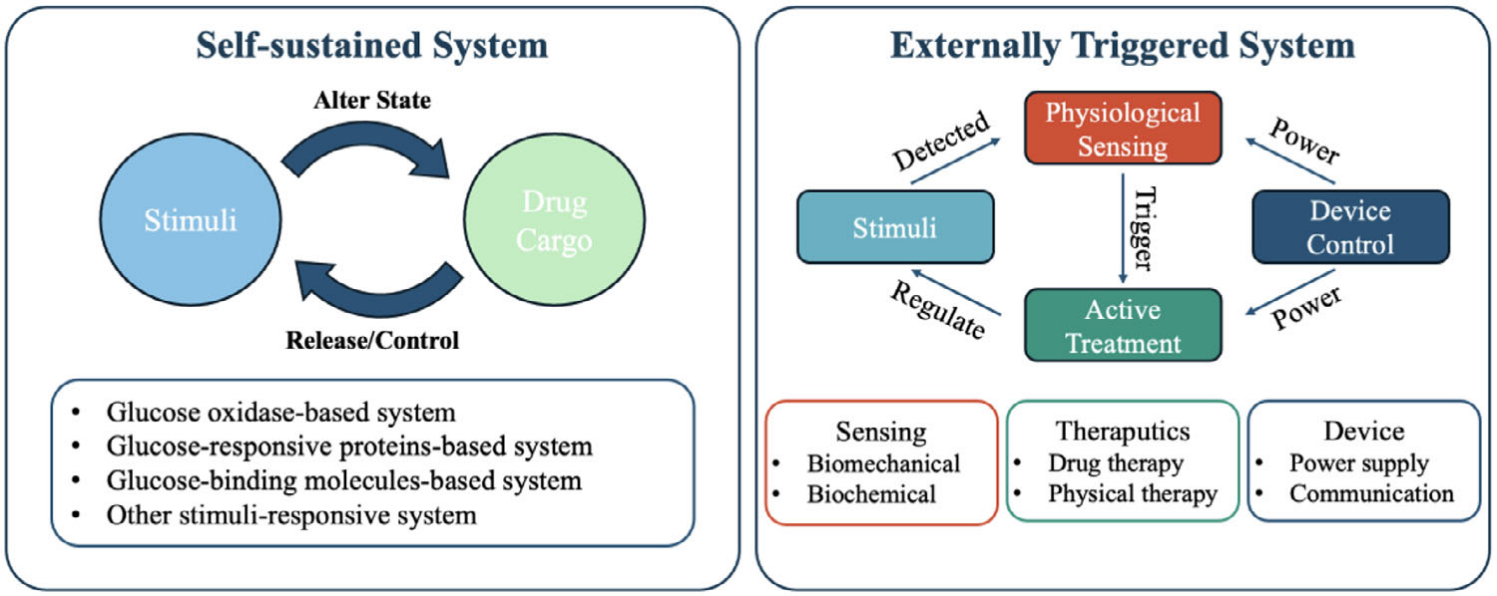
Schematic illustrations of the comparison between self‐sustained system and externally triggered system.

In this review, we will explore recent progress in precision therapy achieved via closed‐loop control systems, classifying them into two primary categories: self‐sustained and externally triggered systems (**Figure** **1**). For self‐sustained systems, bio‐responsive characteristics are crucial to achieving on‐demand and sustainable drug release. This section will explore a variety of stimuli‐responsive materials and the mechanisms that underpin their ability to deliver therapeutics autonomously, along with their applications in managing conditions such as diabetes and cardiovascular diseases. Next, we will examine recent progress in integrated bioelectronics for externally triggered systems, focusing on three critical components: physiological sensing, active treatment, and data analysis for adaptive control. These elements collectively enable real‐time monitoring and dynamic adjustments to therapeutic delivery, ensuring the efficient operation of closed‐loop devices. By offering an in‐depth overview of the potential and limitations of closed‐loop systems in precision therapy, this review highlights their transformative role in advancing personalized medicine and improving patient outcomes.

## 2 Self‐Sustained Closed Loop System

### 2.1 Glucose‐Responsive Drug Delivery

**Figure 2:**
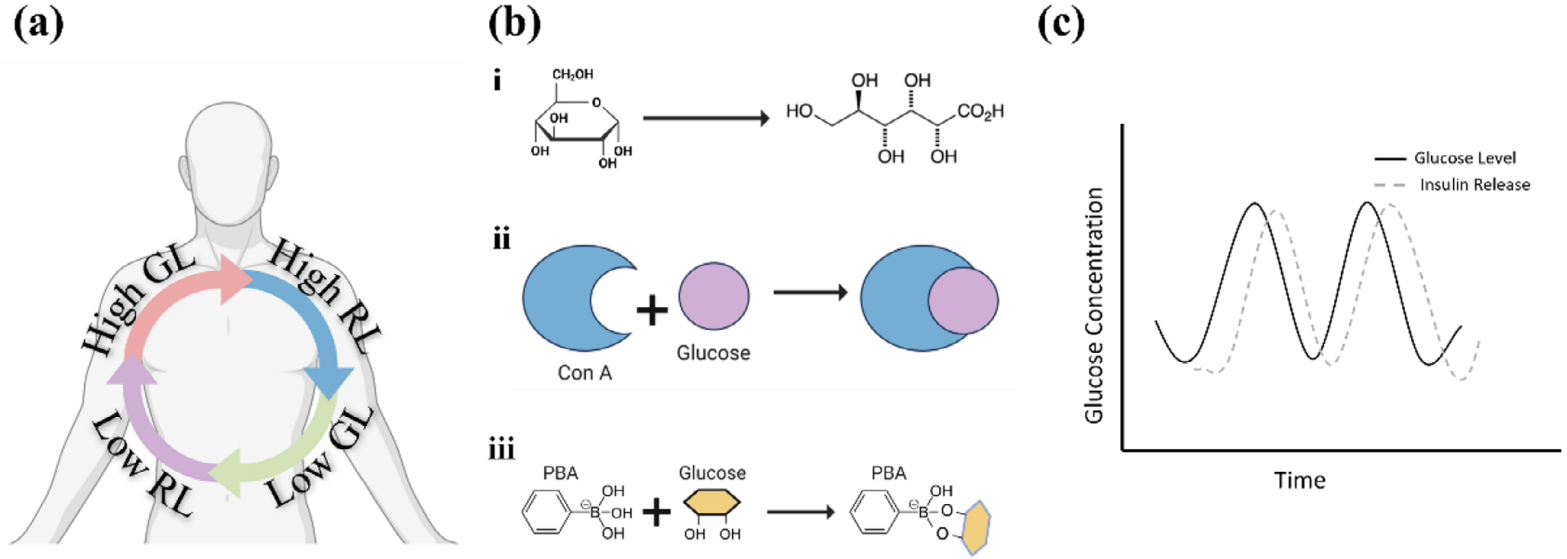
a) Illustration of the feedback loop between glucose level (GL) and drug release level (RL). b) (i–iii) Schematic diagram of the three main glucose‐responsive mechanisms, glucose oxidase‐based system, glucose binding molecule‐based system, and phenylboronic acid‐based system. c) Ideal glucose concentration curve regulated by closed‐loop glucose‐responsive systems. Reproduced with Permission.^[^ ^25^ ^]^ Copyright 2022, The American Association for the Advancement of Science.

Glucose‐responsive drug delivery is a representative example of a self‐sustained closed‐loop system. This system has been widely used to assess its efficacy in providing insulin for type 1 and type 2 diabetes.^[^ ^1^ ^]^ Conventional insulin treatments pose risks of hypoglycemia and inadequate glycemic control when insulin doses fail to match the patient's fluctuating glucose levels.^[^ ^24^ ^]^ In contrast, emerging glucose‐responsive insulin delivery systems offer targeted and more efficient insulin administration by reacting directly to changes in blood glucose. Glucose oxidase, phenylboronic acid, and glucose‐binding molecules are the three primary materials for chemically synthesized glucose‐responsive delivery.^[^ ^2^ ^]^ In the next sections, the three main mechanisms and their corresponding materials design will be discussed, as summarised in **Figure** **2**, illustrating how each contributes to on‐demand insulin release and refined glycemic management

#### 2.1.1 Glucose Oxidase (GOx) Based System

The glucose oxidase (GOx)‐based system controls insulin release by monitoring glucose level changes within the physiological environment, which are driven by the glucose oxidation reaction.^[^ ^1^, ^25^ ^]^ This system undergoes a catalytic reaction, which also has a linear dependence on the glucose concentration. This catalytic process makes the GOx‐based platforms particularly effective for accurate glucose sensing. Specifically, GOx is an enzyme that catalyzes the production of hydrogen peroxide and gluconic acid, from glucose and oxygen as shown in the following reaction:

(1) $$Glucose+O_{2}+H_{2}O\overset{GOx}{\to }Gluconicacid+H_{2}O_{2}$$

Despite its effectiveness in glucose sensing, the GOx‐based enzyme is highly sensitive to environmental factors such as local pH, H_2_O_2_ concentration, and O_2_ level which would fluctuate as glucose concentrations change.^[^ ^26^, ^27^ ^]^ In particular, excess gluconic acid produced during the oxidation process can lower the local pH, potentially leading to enzyme denaturation when the system is incorporated into a polymeric matrix. These factors can ultimately affect both the accuracy and shelf‐life of glucose‐responsive systems.

**Figure 3:**
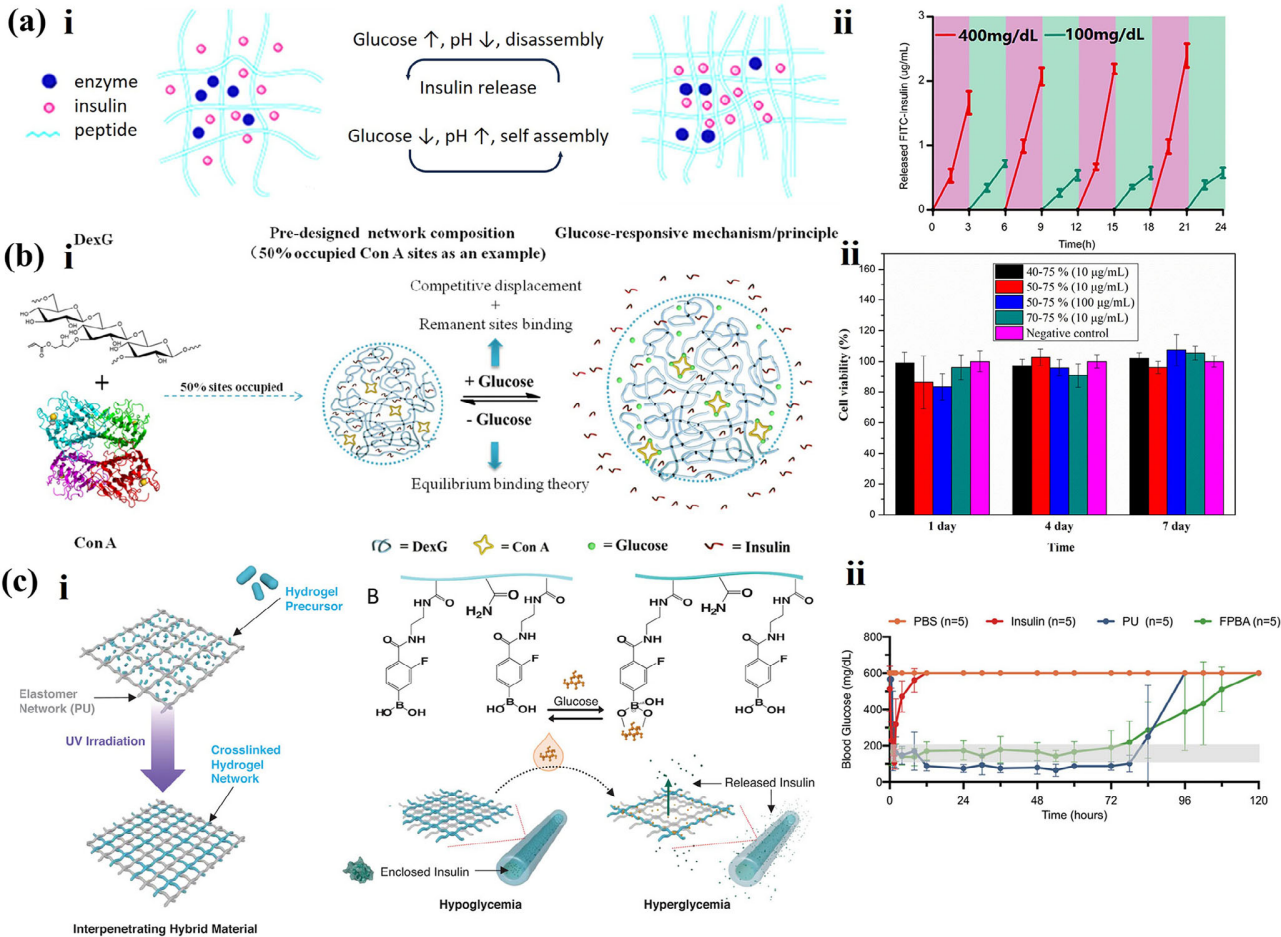
Glucose‐responsive drug delivery. a)‐(i) Schematic diagram of the working principle of GOx‐based glucose‐responsive hydrogel. (ii) In vivo experiment results of pulsatile release profile of FITC‐insulin release when exposed to a hyperglycemic and normal state alternatively. Reproduced with Permission.^[^ ^29^ ^]^ Copyright 2017, Elsevier Ltd. b)‐(i) The fabrication method and mechanism of Con A‐based glucose‐responsive hydrogel. (ii) In vitro experiment results of cytotoxicity evaluation of microgels with concentrations of 10 and 100 µg mL^−1^ by CCK‐8 assay. Reproduced with Permission.^[^ ^34^ ^]^ Copyright 2018, Elsevier B.V. c)‐(i) The glucose‐responsive mechanism for fluorophenylboronic acid (FPBA)‐based glucose‐responsive hydrogel insulin delivery system. (ii) In vivo experiment result of blood glucose levels (BGLs) in diabetic mice after treatment with Phosphate Buffer Saline (PBS), subcutaneous insulin injections (0.05 mg), control non‐glucose responsive cannulas (PU), or glucose‐responsive cannulas (FPBA) loaded with 1.5 mg (100 µL of 15 mg mL^−1^) of insulin. Reproduced with Permission.^[^ ^5^ ^]^ Copyright 2024, Wiley‐VCH GmbH.

To leverage the pH changes induced by gluconic acid, various pH‐responsive mechanisms have been explored. For example, by using polymers with weakly acidic or basic groups in the backbone as hydrogel matrix, the glucose‐oxidation reaction can be transduced into pH‐triggered swelling of the hydrogel, which then controls the insulin release rate and achieve self‐regulation, as illustrated in **Figure** **3**a.^[^ ^28^, ^29^ ^]^ Zeolitic imidazole framework‐8 (ZIF‐8) has also been proposed as a pH‐response system for insulin release.^[^ ^30^ ^]^ These advancements show promising potential for diabetes management.

#### 2.1.2 Glucose‐Binding Proteins Based System

While synthetic hydrogels are commonly used in glucose‐responsive systems, natural alternatives have shown great promise for regulating insulin release. Various naturally occurring proteins, such as glucose‐binding lectin, glucose transporter (Glut), and aptamer, have been exploited for glucose‐responsive drug delivery applications.^[^ ^31^, ^32^, ^33^ ^]^ These proteins can bind to glucose via noncovalent bonding, providing a chemically safe process that does not generate additional byproducts.

Two primary approaches have been proposed in the glucose‐binding systems. The first involves employing carbohydrate moieties that enable glucose‐binding molecules, such as lectins, to form a crosslinking network. In general, Concanavalin A (Con A) is the most widely studied protein from the lectin family for glucose‐responsive systems.^[^ ^35^ ^]^ As depicted in Figure 3b‐(i), glucose‐responsive hydrogels can be produced with a Con A‐polysaccharide matrix, where Con A molecules act as crosslinkers binding different polysaccharide chains together. Cytotoxicity evaluation also confirms the non‐toxicity of Con A under glucose‐responsive system application as shown in Figure 3b‐(ii).^[^ ^34^ ^]^ In the presence of free glucose, the connection between Con A and polysaccharide will be replaced by free glucose, reducing the overall crosslinking density and resulting in swelling of the molecule.^[^ ^4^, ^36^ ^]^ The second approach focuses on modifying insulin to obtain insulin analogs that can reversibly bind to glucose‐binding molecules, such as glycosylated insulin. For instance, one of the commercialized closed‐loop glucose‐responsive system, SmartInsulin®, consist of a complex made between insulin‐dextran conjugate and Con A. Under high glucose concentrations, the glycosylated insulin will be replaced by glucose, leading to simultaneous reduction of glucose and release of insulin.^[^ ^27^ ^]^

Although extensive research has been performed on the above glucose‐responsive systems, there are several limitations to its wider application, including thermal stability, toxicity, and long response time. Researchers have proposed several improved methods to solve these issues. For example, PEGylated Con A can be used instead to mitigate both thermal denaturation and nonspecific electrostatic interactions.^[^ ^37^ ^]^ Alternative glucose‐binding proteins such as Glut and aptamers have also been studied.^[^ ^38^, ^39^ ^]^

#### 2.1.3 Glucose‐Binding Molecules Based System

In contrast to the two previously mentioned mechanisms, phenylboronic acid (PBA) is a small glucose‐binding molecule, that can be easily used under different chemical conditions.^[^ ^3^, ^40^ ^]^ PBA acts as a glucose‐binding molecule that can form a reversible covalent complex with polyol molecules. It is a very stable and durable synthetic molecule, with a physiologically inert chemical structure, making it chemically safe for glucose‐responsive delivery systems. PBA also has high suitability for large‐scale production with low immunotoxicity and denaturisation risks.^[^ ^41^, ^42^ ^]^ Kuivila et al. discovered that PBA can reversibly bind with cis‐1,2‐ or 1,3‐diol structured compounds, through boronate ester formation in an aqueous solution.^[^ ^43^, ^44^ ^]^ In this context, PBA exists in two forms in aqueous solutions: uncharged trigonal planar state and anionic tetrahedral with dynamic balancing with a pKa within the range of 8.2–8.66.^[^ ^45^ ^]^ The uncharged form is relatively hydrophobic, and the charged state is hydrophilic. Upon exposure to glucose, the charged state of PBA forms a stable complex under reversible covalent bonding. Then, by actively attaching to the anionic tetrahedral structure, an equilibrium shift toward the anionic form of PBA is created. This further enhances the hydrophilicity and demonstrates an increased negative charge density.^[^ ^1^, ^27^ ^]^

To date, PBA‐based systems have demonstrated promising preclinical results in both in vitro and in vivo studies. However, translating the system into a clinical application remains challenging. Under physiological conditions, many PBA derivatives exhibit relatively low glucose‐binding affinity, and their affinity is further compromised by higher affinity to certain non‐glucose analytes like fructose and lactate.^[^ ^46^ ^]^ Moreover, the long‐term toxicity and in vivo biocompatibility of PBA moieties remain to be characterized.

To address these limitations, researchers have explored various strategies for constructing glucose‐sensitive sites. For example, glucose‐triggered swelling, dislocations, and replacements have been used to enhance responsiveness, as shown in Figure 3c. With a glucose‐responsive FPBA moiety added into the drug cargo, it is demonstrated that such a design can avoid hypoglycemic episodes, which are commonly observed in the operation of other insulin delivery approaches.^[^ ^5^ ^]^ More recently, Matsumoto et al. have demonstrated the potential of PBA‐based insulin delivery platforms by developing novel PBA compounds, glucose‐responsive gels, and gel‐combined medical devices as drug delivery mechanisms. These technologies incorporate specialized “skin layer” control systems to enable more adaptable and precise insulin administration.^[^ ^44^ ^]^

### 2.2 Other Self‐Sustained System

**Figure 4:**
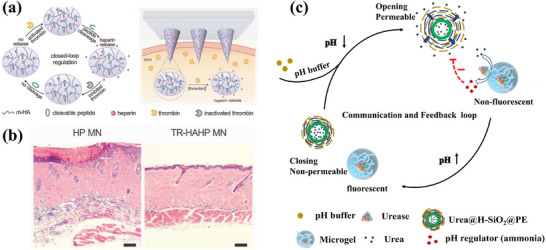
Schematic diagrams representing other self‐sustained close loop systems. a) The mechanism of closed‐loop heparin delivery system in response to thrombin activation. b) Hematoxylin and eosin (H&E)‐stained microscope image of mouse skin after 24h of heparin‐loaded microneedle (HP MN) and thrombin‐responsive hyaluronic acid‐loaded microneedle (TR‐HAHP). Reproduced with Permission.^[^ ^47^ ^]^ Copyright 2018, Elsevier B.V. c) The mechanism of the integrated self‐sustained system, containing stimuli‐responsive capsules and enzyme‐immobilized microgels. Reproduced under the terms of the CC‐BY Creative Common Attribution 4.0 International License (http://creativecommons.org/licenses/by/4.0/).^[^ ^49^ ^]^

Beyond glucose‐responsive systems, various other stimuli‐responsive systems have been explored. One example involves thrombin‐responsive drug delivery platforms for managing cardiovascular conditions. Thrombin is a proteinase that produces insoluble fibrin from soluble fibrinogen in blood coagulation systems. Zhang et al. presented a self‐sustained thrombin‐responsive system for dosing heparin, which is an anticoagulant drug that prevents blockage in blood flow, as illustrated in **Figure** **4**a. As under or over‐dosing heparin can result in dangerous consequences, such as rapid clearance or bleeding complications in the body, feedback‐controlled drug delivery is crucially needed. In this work, a thrombin‐cleavable peptide is used when conjugating heparin (HP) to hyaluronic acid (HA), forming the TR‐HPHA matrix. When thrombin is activated, the peptide will be cleaved and trigger the release of the drug. By applying this matrix onto the microneedle patch, the device will be able to respond to an increase in thrombin level and release the corresponding dosage of the anticoagulant drug. Therefore, a closed‐loop device is obtained.^[^ ^47^, ^48^ ^]^ This study has further conducted a diabetic mouse model to demonstrate in vivo performance from glucose‐responsive insulin delivery. The system demonstrated precise, self‐regulated insulin release in response to glucose fluctuations over several days. Importantly, immune profiling indicated minimal inflammatory response and no evidence of systemic toxicity, highlighting the system's biocompatibility, as shown in Figure 4b. These findings underscore the clinical promise of autonomous therapeutic platforms that adapt to dynamic physiological conditions with high temporal fidelity.

Similarly, as shown in Figure 4c, Zhou et al. also suggested a homeostatic, self‐sustainable, pH‐responsive closed‐loop system. This system is a dual colloidal system with a pH‐responsive capsule (Urea@H‐SiO_2_@PE) and an enzyme‐integrated microgel. As pH decreases, urea is released from the capsule and incorporated with the enzyme, urease, to transform into ammonia, which increases the pH level. This then limits the release of urea, leading to a self‐regulatable closed‐loop system, which can be used as a model for potential applications.^[^ ^49^ ^]^

## 3 Externally Triggered Closed Loop System

**Figure 5:**
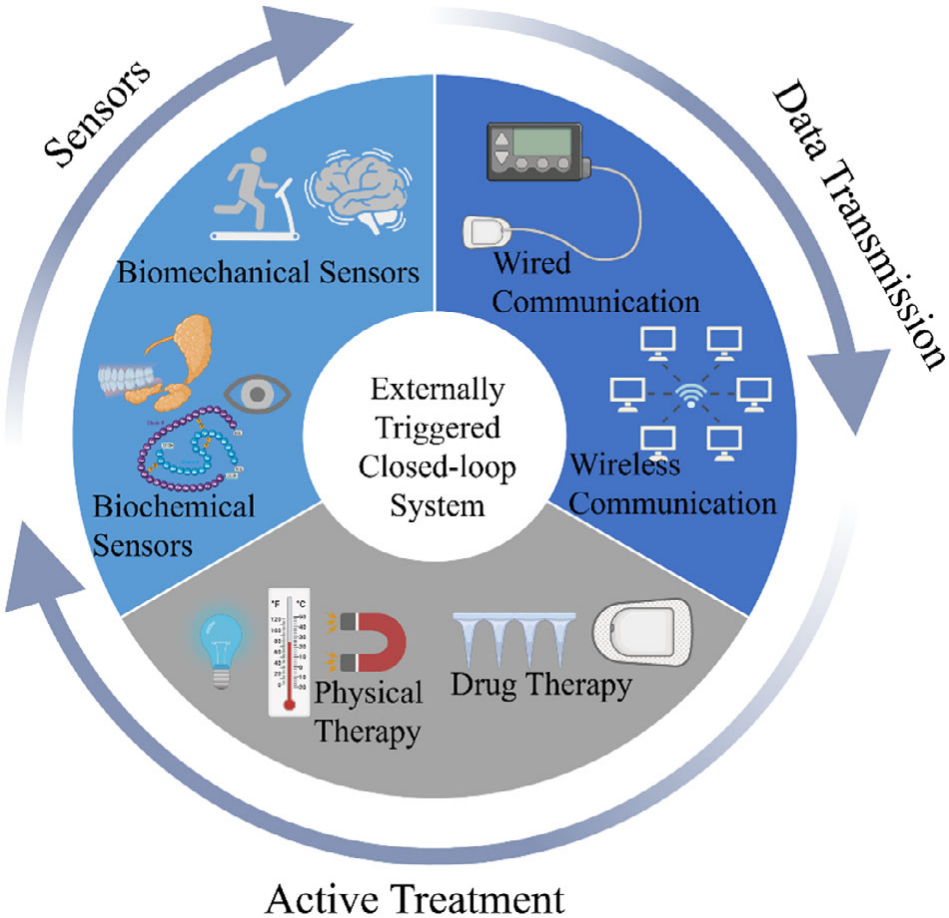
Schematic diagram representing main components of externally triggered closed‐loop systems.

While self‐sustained systems offer advantages such as simplicity and full biodegradability, they are limited by the types of physiological signals suitable for triggering them. Externally triggered systems address this constraint by relying on external inputs to initiate therapeutic actions, enabling enhanced precision and broader applicability. As illustrated in **Figure** **5**, these systems are versatile and can be employed across a wider range of diseases, making them especially attractive for targeted therapies in diverse medical contexts.

Externally triggered closed‐loop systems comprise three primary components: physiological sensing, active treatment, and data analysis for adaptive control. First, the system's sensors detect local environmental changes, ranging from biomechanical signals (e.g., pressure or strain) to biochemical markers (e.g., pH levels). The collected data is then transmitted to the electronic control unit, which processes the information and determines the appropriate therapeutic actions, such as drug release or electrical stimulation. Finally, based on the sensor feedback, the therapeutic unit executes the on‐demand treatment, ensuring precise and timely therapeutic intervention by releasing the drug or providing the necessary stimulation. This seamless integration of sensing, processing, and therapy enables highly accurate and responsive treatment capabilities of externally triggered closed‐loop systems.

In this section, we will explore the mechanisms of physiological sensing and monitoring, focusing on both biomechanical and biochemical signals. Next, we will discuss various therapeutic approaches, including drug delivery and physical therapy. Additionally, we will examine different communication strategies for data analysis, which are critical to the effective operation of these systems.

### 3.1 Physiological Sensing and Monitoring

A sensing unit is a critical component in externally triggered closed‐loop systems, designed to detect changes in physiological signals and convert them into electrical signals for further processing. The choice of suitable sensors depends on the specific therapeutic requirements and the targeted medical condition, ensuring accurate and efficient monitoring. Biosensors are typically categorized into two main types based on the nature of the signals they detect. Biomechanical sensors measure physical changes in the human body, such as pressure, strain, and temperature, providing insights into mechanical and thermal variations. Biochemical sensors, on the other hand, focus on detecting metabolite levels in biofluids like sweat, interstitial fluid, tears, and saliva, enabling the identification of critical biomarkers for disease management and treatment.^[^ ^50^ ^]^

#### 3.1.1 Biomechanical Signals

Biomechanical motions, such as limb movements, cardiovascular contractions, and pulses in blood vessels, generate strain or pressure that can be detected via various sensory techniques.^[^ ^51^ ^]^ Pressure sensors are commonly employed to monitor these motions, and their sensing mechanisms are broadly categorized into capacitive, piezoresistive, and piezoelectric sensors. Capacitive sensors are devices that measure changes in capacitance to detect physical or environmental changes. These sensors offer lower power consumption with a faster response period, making them particularly suitable for real‐time applications. In contrast, piezoresistive sensors monitor the differences in electrical resistance arising from external pressure, offering reliable performance for many biomedical applications.^[^ ^52^ ^]^ Piezoelectric sensors are capable of generating electrical voltages when mechanically displaced, functioning as self‐powered sensors. These sensors are widely used for many biomedical fields: including monitoring eye motion,^[^ ^53^, ^54^ ^]^ heart rate,^[^ ^55^, ^56^, ^57^, ^58^ ^]^ blood pressure,^[^ ^57^, ^59^ ^]^ and respiration rate.^[^ ^60^ ^]^ Different parts of the human body produce distinct pressure levels, where intrabody pressure, such as intraocular or intracranial, is relatively low (<10 kPa), cardiovascular and respiratory pressures tend to be moderate (<100 kPa), and forces from body weight can exceed 100 kPa.^[^ ^61^ ^]^ However, since many biomechanical motions produce extremely small forces, sensor sensitivity and accuracy are critical. Recent studies suggest combining piezoelectric and triboelectric mechanisms to create energy‐harvesting devices capable of detecting subtle facial movements and blinking,^[^ ^62^ ^]^ enabling differentiation between voluntary and involuntary actions and providing more efficient, direct feedback.

**Figure 6:**
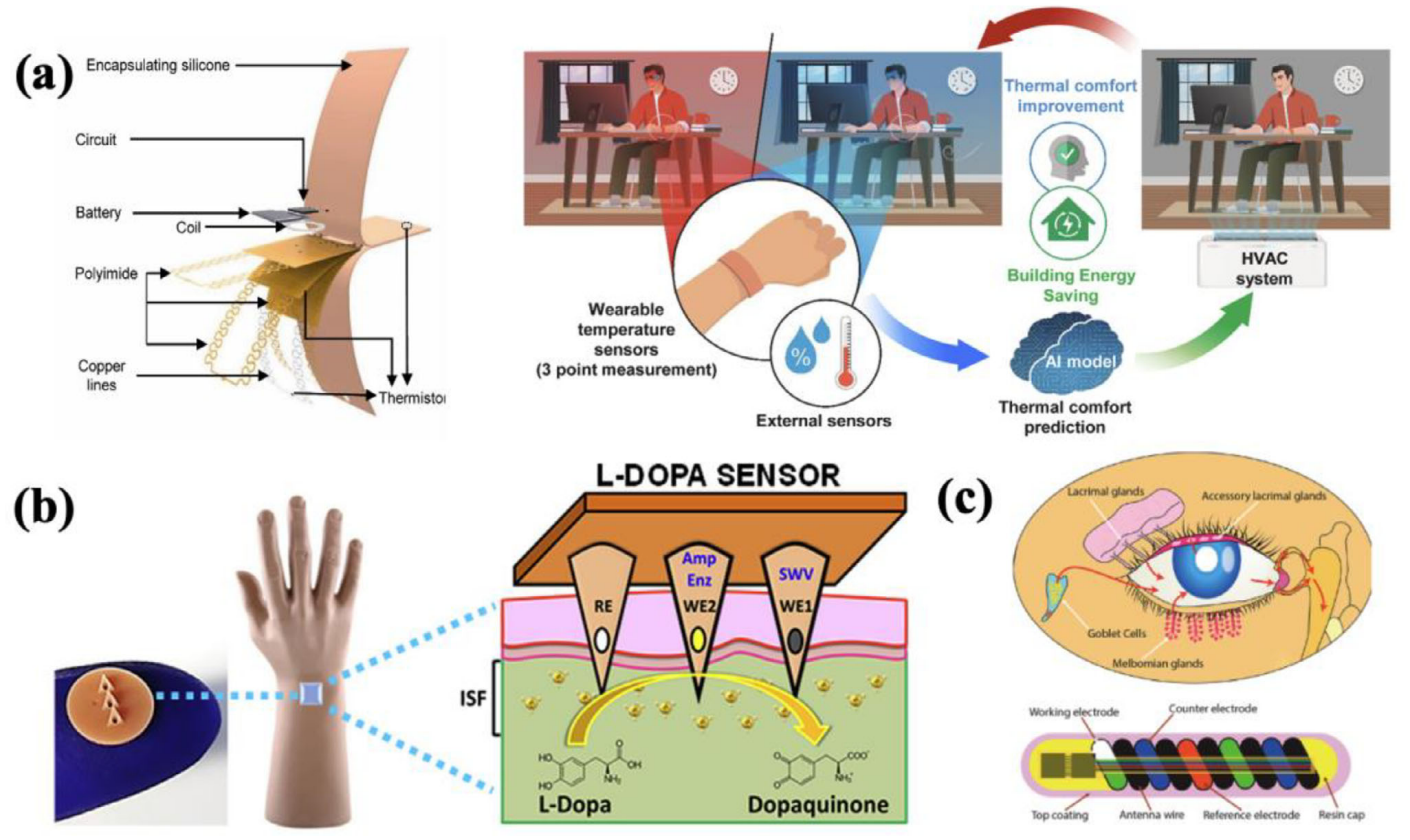
Examples of physiological sensing systems. a) Schematic diagram of the overall operation principle of the temperature regulation system. Reproduced with Permission.^[^ ^63^ ^]^ Copyright 2022, Elsevier B.V. b) Schematic diagram of microneedle sensor for L‐Dopa detection. Reproduced with Permission^[^ ^17^ ^]^ Copyright 2016, WILEY‐VCH Verlag GmbH & Co. KGaA, Weinheim. c) Schematic diagram of the NovioSense tear glucose sensor and the structure of the tear fluid production. Reproduced under the terms of the CC‐BY Creative Common Attribution 4.0 International License (http://creativecommons.org/licenses/by/4.0/).^[^ ^64^ ^]^

Temperature sensors also play a vital role in closed‐loop systems by tracking changes in both ambient and physiological temperature. These sensors often work together with thermal heaters to create a feedback loop that maintains an optimal temperature level. As shown in **Figure** **6**a, a study by Cho et al. introduced wireless, AI‐enabled thermal comfort sensors capable of accurately predicting an occupant's thermal comfort.^[^ ^63^ ^]^ This human‐centric control system offers a new solution that improves the occupant's thermal comfort and provides significant energy savings.

#### 3.1.2 Biochemical Signals

Biochemical sensors detect chemical changes in real time, offering a continuous stream of data for closed‐loop healthcare systems. Commonly monitored biomarkers, such as glucose, lactate, and electrolytes, are found in various biofluids, including sweat, interstitial fluid (ISF), saliva, and tears. These biosensors are typically integrated into wearable formats and have been used as a non‐invasive approach for capturing physiological information from biofluids. For example, in contrast to self‐sustained systems that depend on a limited range of bio‐responsive materials, bioelectronics‐based solutions can achieve on‐demand insulin delivery by leveraging real‐time biochemical sensing, thereby enhancing glucose control and reducing hypoglycaemic risks. This approach provides higher accuracy and allows more automation in insulin delivery based on continuous glucose monitoring for diabetes management.^[^ ^65^ ^]^

Sweat is one of the most accessible biofluids as it can be secreted from the eccrine glands and easily collected from the skin surface. Its straightforward accessibility makes sweat a suitable candidate for biosensing and disease detection. Sweat contains several measurable biomarkers, such as small lipophilic analytes, electrolytes, lactate, and urea,^[^ ^66^, ^67^, ^68^ ^]^ and studies have shown a strong correlation between blood and sweat concentrations of lipophilic compounds.^[^ ^69^ ^]^ Sweat can also measure other analytes, such as electrolytes, including sodium, potassium, and chloride. Sodium is particularly useful for detecting electrolyte imbalance,^[^ ^68^ ^]^ while lactate and urea levels help detect muscle fatigue or kidney dysfunction.^[^ ^70^ ^]^

ISF is another critical biofluid rich in biomarkers, including proteins, small‐molecule metabolites, and RNA, making it highly sensitive to local tissue changes.^[^ ^71^ ^]^ As an epidermally accessible biofluid, ISF can be collected continuously and analyzed via body‐interfacing devices.^[^ ^72^, ^73^ ^]^ Two main sensor types have been employed for ISF‐based monitoring: microneedle and reverse‐iontophoresis sensors. Microneedle‐based sensors provide stable, minimally painful measurements by directly penetrating inter‐tissued space.^[^ ^74^ ^]^ For example, Goud et al. demonstrated a wearable chemical sensing platform based on a microneedle electrode array for continuous monitoring of the antiparkinsonian drug L‐Dopa, as shown in Figure 6b.^[^ ^17^ ^]^ This system offers the potential for closed‐loop Parkinson's disease management with optimal L‐Dopa dose by combining L‐Dopa pumps with dose‐automation algorithms. Reverse iontophoresis, on the other hand, uses electro‐osmotic ion flow to detect biomarker information from the skin by applying a potential difference.^[^ ^75^, ^76^ ^]^

Saliva‐based sensors offer another attractive, non‐invasive monitoring option, given saliva's ready availability and its correlation with blood concentrations of numerous analytes.^[^ ^77^ ^]^ Saliva can reflect various key physiological information, including hormonal, immunological, and metabolic conditions.^[^ ^78^ ^]^ and is commonly used to measure glucose, hormones, and lactate. Valdes‐Ramirez et al. proposed a mouth guard‐based system with a printable enzymatic biosensor to continuously monitor saliva lactate.^[^ ^79^ ^]^ This system allows stable and sensitive lactate response in saliva and provides potential applications in‐mouth saliva‐based sensors, such as monitoring pH, dehydration detection, and immune response.

Tear is also an important biomarker source. Compared to human skin, the human eye is softer and more sensitive to pain, making continuous tear biosensing particularly challenging. While contact lens‐based sensors are user‐friendly, a lack of long‐term clinical data raises concerns about potential side effects.^[^ ^80^ ^]^ Kownacka et al. proposed a wearable capsule‐based sensor for continuous tear glucose monitoring (Figure 6c). The device is coated with polysaccharide‐based hydrogel, providing high flexibility, and enabling painless, unobtrusive tear collection.^[^ ^64^ ^]^ This approach addresses the limitations of contact lens‐based sensors and shows a promising solution for long‐term tear biosensing.

### 3.2 Active Treatment

In a closed‐looped system for precision therapy, once the sensor detects abnormal physiological conditions, active treatment is needed to rapidly alleviate symptoms or regulate normal body function. Active treatments can be in various forms, including drug therapy, physical therapy, or a combination of both. The performance of active treatments is typically evaluated through several common criteria: effective alleviation of symptoms, rapid response time when receiving feedback, and the precision of the treatment. In this section, recent progress in active treatment approaches will be summarised and discussed.

#### 3.2.1 Drug Therapy

Drug therapy is the most employed method of active treatment in closed‐loop systems. Its working principle is relatively straightforward, involving the direct release of the corresponding drug into the body. In such a system, a pre‐loaded drug will be on demand and released into the body, with dosage controlled in real time according to the patient's condition.^[^ ^81^ ^]^ Despite its simplicity, traditional electrically controlled drug delivery system still faces some challenges in miniaturization, drug protection, and well‐controlled release process.

**Figure 7:**
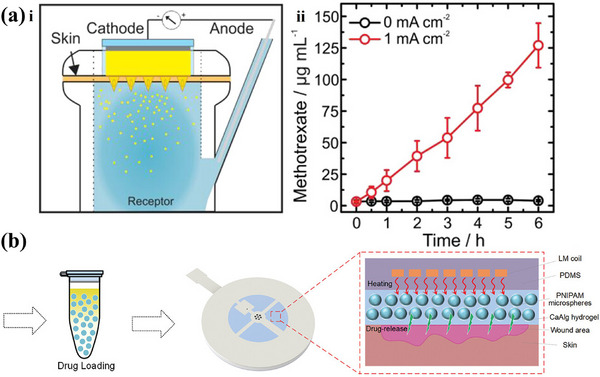
Examples of active treatment via drug delivery. a) (i) Schematic diagram of the setup for the MTX‐loaded microneedle patch, (ii) the drug release rate in the microneedle patch. Reproduced with Permission.^[^ ^82^ ^]^ Copyright 2023, American Chemical Society. b) Schematic diagram of the drug‐loaded hydrogel in the microfluidic system. Reproduced with Permission.^[^ ^83^ ^]^ Copyright 2023, Wiley‐VCH GmbH.

Several innovative drug delivery strategies have been introduced to address these challenges. Parrilla et al. employed a microneedle patch design to realize high‐precision on‐demand delivery of Methotrexate (MTX), as depicted in **Figure** **7**a. In this design, the MTX drug is loaded into hydrogels and left in contact with an Ag/AgCl cathode. When current is applied to the system, chloride ions will be released to promote the outward migration of MTX. This microneedle patch enables stable, linear delivery of MTX within a minimal area of 5mm^2^.^[^ ^82^ ^]^ Similarly, Ge et al. devised an intelligent wound dressing (IWD) for chronic wound treatment, as shown in Figure 7b, by incorporating a liquid metal heating coil into a thermoresponsive Poly(N‐isopropyl acrylamide) (PNIPAM) hydrogel. Upon heating, the pre‐loaded antibiotic cefazolin can be fully released within 20 min, demonstrating the potential for efficient, rapid, and localized drug delivery in wound care.^[^ ^83^ ^]^ Also utilizing the drug delivery capability of hydrogels, Qin et al. have integrated a flexible triboelectric nanogenerator (TENG) into the device and proposed an innovative self‐powered wearable triboelectric stimulator that allows long‐term consistent release of curcumin nanoparticles for wound healing.^[^ ^84^ ^]^

#### 3.2.2 Physical Therapy

Physical therapy offers alternative treatment approaches by utilizing electrical, optical, magnetic, or mechanical modulators to regulate physiological processes. These therapies are particularly valuable in addressing conditions involving neural activity, organ function, or biomolecule production. The interaction between actuators and organ tissues critically influences the effectiveness of these treatments.^[^ ^23^ ^]^

Light‐based therapy, also known as phototherapy, is a widely used treatment method that can be categorized into three main types: photodynamic therapy (PDT), photothermal therapy (PTT), optogenetics, and near‐infrared photoimmunotherapy. PDT utilizes a photosensitizer that responds to specific wavelengths of light to produce reactive oxygen species in the presence of molecular oxygen, leading to the destruction of targeted tissues.^[^ ^85^, ^86^ ^]^ PTT, on the other hand, converts light energy into heat. The localized heat generated can be employed to selectively damage targeted cells and tissues without harming the surrounding healthy areas. Additionally, PTT can increase glutathione consumption and provide anti‐cancer treatment. Recent studies have also combined PDT and PTT to develop novel therapeutic approaches, significantly improving their impact on breast cancer treatment by leveraging the complementary mechanisms of these two therapies.^[^ ^87^, ^88^, ^89^ ^]^ Optogenetics, meanwhile, is a stimulation method based on expressing light‐sensitive proteins in neuronal cell membranes, enabling either transdermal stimulation or tissue‐selective induction.^[^ ^90^ ^]^ Srinivasan et al. proposed a closed‐loop functional optogenetic stimulation system designed to control ankle joint position in murine models. The system utilizes joint angle measurements as feedback signals to adjust stimulation dynamically.^[^ ^10^ ^]^ This study establishes a foundation for developing closed‐loop optogenetic neuromuscular stimulation therapies aimed at precise peripheral limb control. Near‐infrared photoimmunotherapy (NIR‐PIT) is an emerging targeted cancer treatment that combines antibodies and NIR light. Unlike conventional PDT, NIR‐PIT achieves highly selective cancer cell destruction with minimal collateral damage to the surrounding healthy tissues.^[^ ^91^ ^]^ Closed‐loop control could enhance NIR‐PIT by adjusting light dose or timing, based on the feedback from the tumor. By incorporating these light‐based therapies with sensors, such as optical, thermal, or biochemical, more dynamic, regulated light delivery for safer and more effective treatment can be achieved. Mechanical actuators, which generate motion by controllably exerting force and deformation, are less frequently incorporated into biomedical closed‐loop systems but play a crucial role in assisting or replacing natural body movements when muscle dysfunctions occur, such as in cases of heart failure or restenosis. To operate effectively inside the human body, mechanical actuators must be soft, flexible, and biocompatible, while at the same time being able to provide sufficient force to replace original muscle function.^[^ ^92^ ^]^ Recently, various innovative materials and device designs have been explored. For instance, Payne et al. proposed a novel soft robotic device based on a pneumatic actuator for the regulation of heart failure. Several McKibben actuators, one of the common pneumatic muscles, are wrapped around heart ventricles with elastic sleeves to replicate natural cardiac contraction and relaxation cycles.^[^ ^6^ ^]^ Similarly, the respiratory system, like the cardiovascular system, involves significant mechanical motions, and its dysfunction can lead to severe and life‐threatening conditions. To address respiratory issues, Zhang et al. developed a soft robotic system as a solution for patients with respiratory difficulties. The proposed design features a soft wearable robot with an inspiration module that applies in vitro negative pressure to assist the outward expansion of the rib cage, and an expiration module can apply positive pressure to squeeze the diaphragm upward. Compared to traditional respiratory regulation systems such as “iron lung”, this soft respiratory assistive robot reduces the risk of lung injury by more closely resembling the natural respiration process and provides flexibility and wearability that could greatly expand the application of ventilation devices.^[^ ^93^ ^]^

Another mechanical modality in closed‐loop systems is microbubble‐enhanced focused ultrasound (MB‐FUS), particularly when combined with immunotherapy. In MB‐FUS, intravenous microbubbles circulate through the target tissue, and ultrasound bursts cause them to oscillate, increasing tissue permeability and stimulating immune signaling. Closed‐loop control in MB‐FUS systems uses real‐time acoustic feedback to optimize safety and efficacy. Lee et al. developed a closed‐loop MB‐FUS platform for glioblastoma treatment.^[^ ^94^ ^]^ This system can effectively modulate blood‐brain barrier (BBB) permeability and improve the delivery of anti‐cancer agents while promoting immune responses in the tumor microenvironment. The findings highlight the potential of closed‐loop MB‐FUS in brain cancer immunotherapy and its applicability to other neurological diseases.

Electrotherapy has also gained much attention recently as a form of physical therapy, particularly for wound healing and tissue repair.^[^ ^95^ ^]^ Compared to traditional treatments, electrical stimulation can reduce the risk of bacterial infection without the risk of antibiotic resistance, and at the same time expedite tissue regeneration.^[^ ^96^ ^]^ Kim et al. developed a customized wound patch for advanced tissue regeneration with an electric field system, where a computer‐vision‐based system is employed to guide the real‐time printing of the patches. The closed‐loop feedback system between wound scanning and device fabrication enables efficient wireless power transmission and improved electrical stimulation accuracy.^[^ ^97^ ^]^ The application of closed‐loop controlled systems in electrotherapy is also widely explored for the treatment of cardiovascular and neurological diseases. In these areas where electrical stimulation‐based treatments, such as pacemakers and deep brain stimulation (DBS), are already developed as prevailing strategies, the integration of closed‐loop control systems allows on‐demand stimulation and better handling of complex situations.^[^ ^8^, ^98^, ^99^ ^]^

### 3.3 Data Transmission and Adaptive Control

Continuous monitoring of health conditions through biosensors is critical for precise therapeutic control, as it enables real‐time dosage adjustments and ensures effective on‐demand treatment. Stable and efficient data transmission, both internally and to external devices, is crucial for maintaining up‐to‐date physiological information and guiding therapeutic interventions. Two primary communication approaches are commonly used: wired and wireless systems, each with its advantages and limitations.

#### 3.3.1 Wired Communication System

Wired communication systems provide high stability and effectiveness by directly connecting the biomedical device to an external apparatus, ensuring efficient data transfer and reducing signal interference. Additionally, wired setups can offer a reliable power supply, thereby facilitating continuous operation. Common applications include neonatal monitors, cardiac monitors, and EEG machines. However, wired systems come with limitations: the cables may cause discomfort or skin irritation and could be damaged when exposed to water. Additionally, they are prone to interruptions when placed near other electrical appliances. For instance, in blood‐pressure monitoring systems, wired connections can lead to catheter knotting, valvular damage, inflection, and data distortion from motion artifacts.^[^ ^7^ ^]^ Consequently, wired systems are typically limited to temporary use for non‐mobile patients in controlled environments, such as hospitals, clinics, and research laboratories.

#### 3.3.2 Wireless Communication System

Wireless communication systems enhance flexibility in data transmission by eliminating the need for physical connections with wires and cables, thereby reducing patient discomfort, and enabling more convenient monitoring and control. These systems can be divided into three main communication methods: Bluetooth Low Energy (BLE), Near Field Communication (NFC), and hybrid NFC‐BLE.

**Figure 8:**
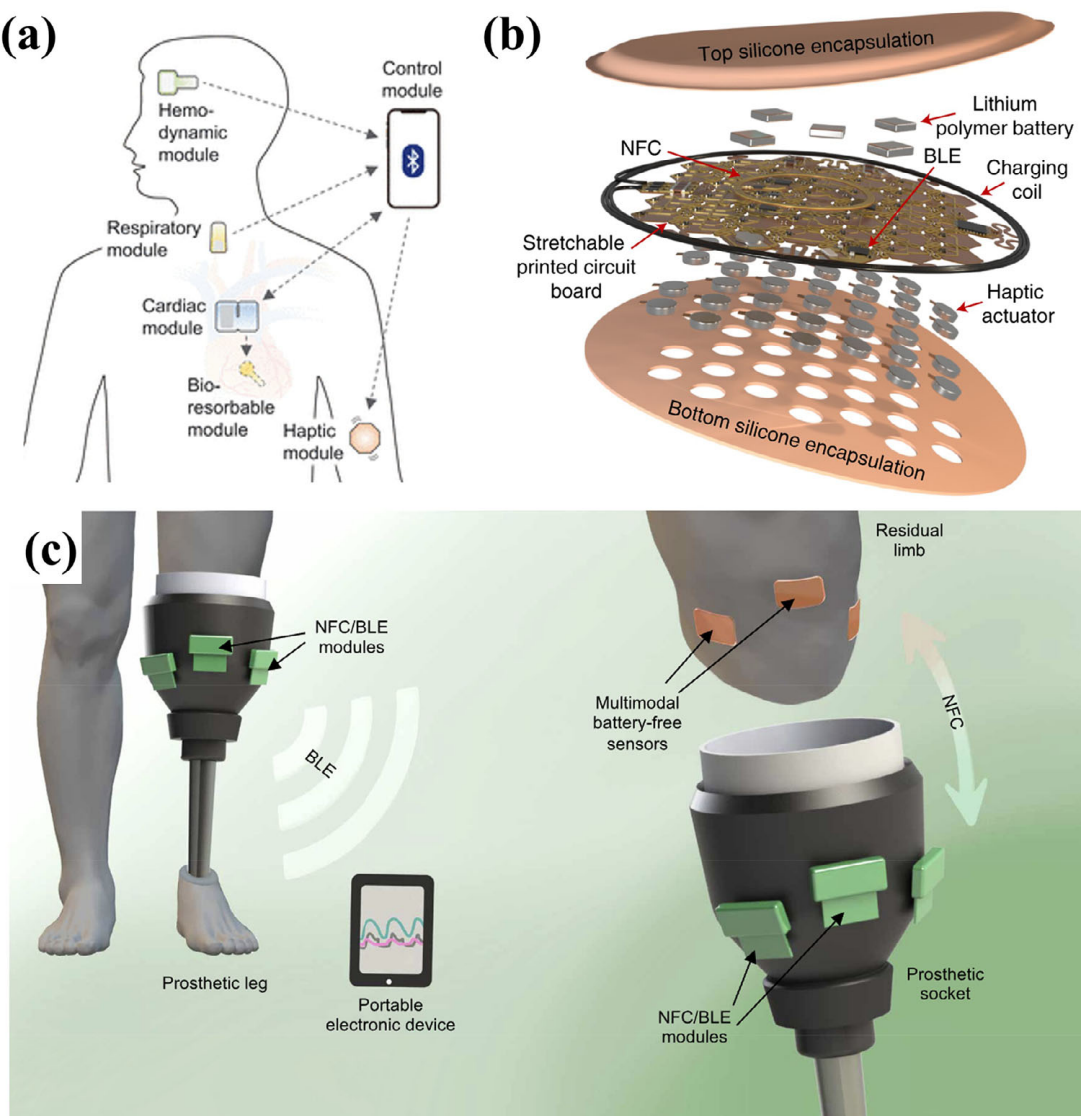
Examples of wireless communication systems. a) Schematic diagram of a wireless device for autonomous electrotherapy. BLE communication is used for providing patient feedback and obtaining pacing functions. Reproduced with Permission.^[^ ^8^ ^]^ Copyright 2022, The American Association for the Advancement of Science. Examples using hybrid NFC‐BLE communication systems. b) Schematic diagram of the exploded view of the electrical components inside the haptic interface device. Reproduced with Permission.^[^ ^110^ ^]^ Copyright 2022, The Author(s), under exclusive license to Springer Nature Limited. c) Schematic diagram of the overall system for wireless multimodal sensing between a residual limb and a prosthesis. Reproduced with Permission.^[^ ^111^ ^]^ Copyright 2020, The American Association for the Advancement of Science.

Among state‐of‐the‐art biomedical devices, Bluetooth low energy (BLE) communication is widely used in wearable devices due to its low power consumption and suitability for prolonged battery life. It is commonly employed in fitness trackers, smartwatches, and continuous glucose monitors.^[^ ^100^, ^101^, ^102^ ^]^ For example, Choi et al. proposed a body‐integrated device for autonomous electrotherapy, which transmits physiological data to the control module via BLE communication, as shown in **Figure** **8**a. This approach enables customizable control modules that support a wide range of device types, offering improved flexibility and functionality.^[^ ^8^ ^]^

NFC, a type of radio frequency identification (RFID), allows secure, short‐range communications for a few centimeters.^[^ ^103^ ^]^ It is commonly used in applications such as contactless payment systems, or other secure data transfer between devices and smartphones.^[^ ^104^ ^]^ Communication modules based on these conventional wireless communication strategies are commercially available and can be readily integrated into implantable biomedical devices.^[^ ^105^, ^106^, ^107^, ^108^ ^]^ For example, Xiong et al. developed a wireless wound infection sensor based on NFC wireless data transmission. In the proposed device, the integrated NFC module allows wireless power transmission to the LC sensing module, while electronic signals from the DNA hydrogel electrode can be easily read out from a smartphone at the same time.^[^ ^109^ ^]^

Hybrid NFC‐BLE devices have been developed, incorporating a single wireless inductive module that simultaneously harvests power at the NFC frequency (13.56 MHz) and transmits measurements to an external interface via the BLE protocol. This innovative design is attracting significant interest in the biomedical field. For instance, as demonstrated in Figure 8b, Jung et al. presented a wireless haptic interface for skin sensations. This interface aims to realistically convey sight, sound, pressure, and other information to mimic the natural feeling of touch. With this design, the device can capture 700 mW of power at a distance of 50 cm from the transmission antenna. This enables activation of the system and full power operation without the need for any battery component.^[^ ^110^ ^]^ Additionally, Kwak et al. integrated a hybrid NFC‐BLE system into a wireless prosthetic limb, as shown in Figure 8c,^[^ ^111^ ^]^ using NFC to gather temperature data and BLE to transmit it to an external unit for real‐time monitoring. This dual wireless design eliminates the risks associated with batteries near skin interfaces, such as incompatibility, size, bulk, and health concerns, which also enables long‐range operations through the BLE communication method.^[^ ^7^, ^112^ ^]^

However, despite their advantages, hybrid NFC‐BLE systems face several challenges. Energy harvesting efficiency significantly decreases in deep tissue applications due to electromagnetic signal attenuation, limiting their use to superficial or surface‐level devices. Moreover, the effective communication range of NFC is limited (usually only a few centimeters), while BLE, although it can provide a longer range, is more sensitive to antenna design and radio frequency interference.^[^ ^111^ ^]^ Therefore, the hybrid NFC‐BLE architecture requires careful design of antenna layout and reader position in practical applications to avoid signal loss or interference. The interference between the two communication systems can disrupt data integrity and system reliability in complex clinical environments. Addressing these limitations is critical for the broader adoption of hybrid NFC‐BLE solutions in implantable or densely networked biomedical applications. The hybrid NFC‐BLE offers a highly efficient solution for wireless communication in next‐generation biomedical devices.

#### 3.3.3 Power Supply

Ensuring a reliable and stable power supply is one of the primary design considerations in closed‐loop systems, as different power sources can significantly impact device functionality, efficiency, and safety. Three main power supply strategies have emerged for wearable and implantable devices: onboard batteries, battery‐less designs, and battery‐free systems.

An onboard battery remains a common choice, providing a stable energy source and minimizing the risk of power fluctuations. However, the need for periodic battery replacement adds complexity to treatment. A classic example is the implantable cardioverter‐defibrillator (ICD), which periodically requires a battery change due to depletion.^[^ ^113^ ^]^ These devices must supply both a constant level of electricity for monitoring and a high‐intensity pulse for delivering cardiac shocks. Accordingly, ongoing research focuses on enhancing battery longevity and stability. Among commercialized devices, lithium‐ion batteries remain the most widely used due to their high energy density. However, due to the safety and stability concerns of lithium‐ion batteries, alternatives such as zinc‐manganese batteries are also proposed for achieving stable performance and reducing the risk of thermal runaway.

**Figure 9:**
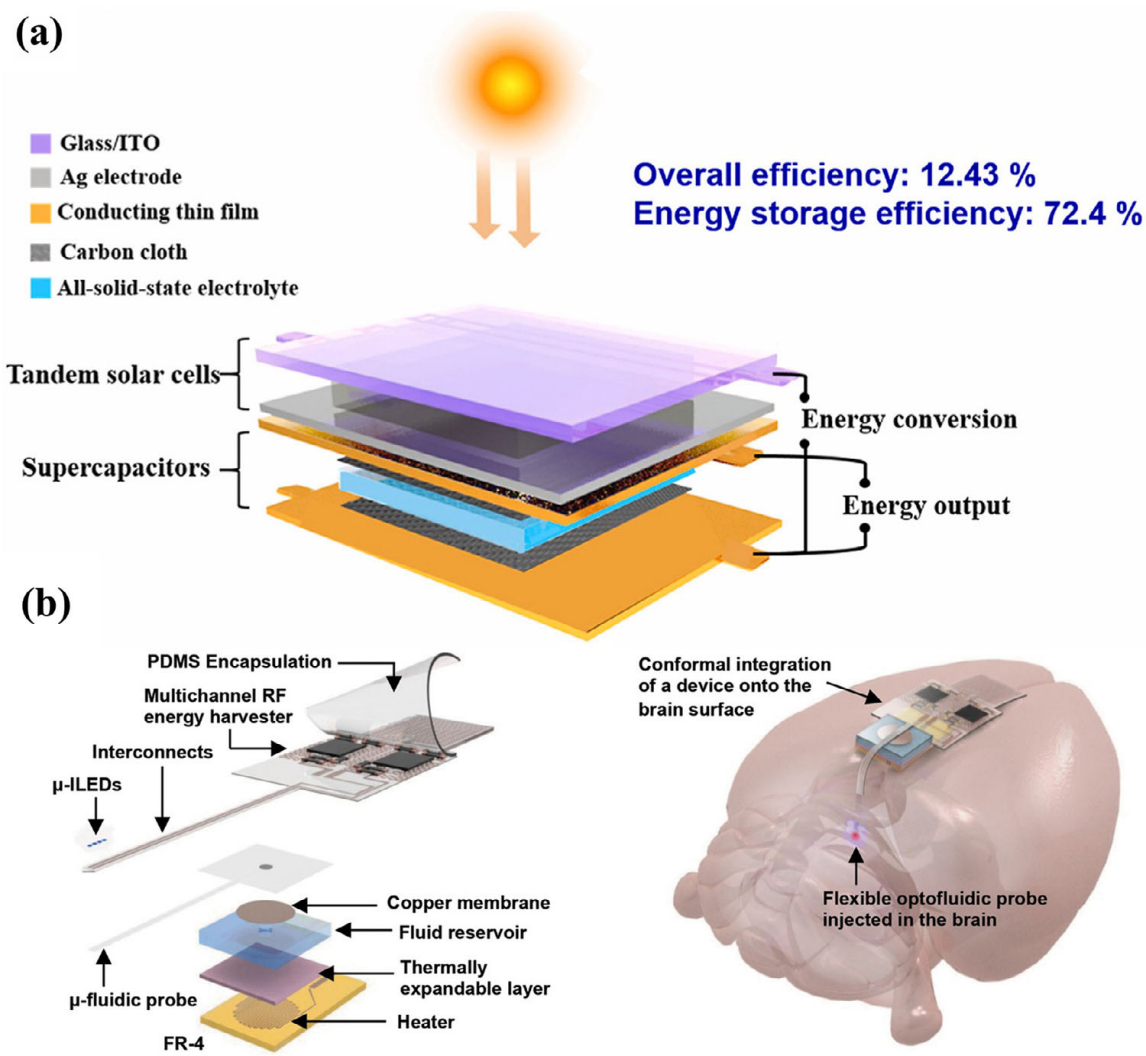
Power supply modules for closed‐loop biomedical devices. a) Diagram of tandem solar cells energy harvesting unit in combination with supercapacitor energy storage unit. Reproduced with Permission.^[^ ^115^ ^]^ Copyright 2020, Elsevier Ltd. b) Diagram of wireless RF power transmission modules configuration in the application of optofluidic brain monitoring. Reproduced with Permission.^[^ ^9^ ^]^ Copyright 2017, WILEY‐VCH Verlag GmbH & Co. KGaA, Weinheim.

Besides widely used onboard battery design, battery‐less and battery‐free power supplies have also been explored as emerging solutions. Battery‐less and battery‐free differ in the input and output mechanisms. Battery‐less powered devices harvest energy from the environment, such as solar power, and kinetic energy, and store energy in capacitors to be used for power transfer within closed‐loop systems.^[^ ^113^ ^]^ A typical example is the superconductor, which can deliver energy at a controllable rate while retaining high power density and long lifetime.^[^ ^114^ ^]^ Zhu et al. innovatively integrated supercapacitors with perovskite solar cells and created a wireless self‐charging power pack, depicted in **Figure** **9**a, leveraging supercapacitors’ rapid storage properties.^[^ ^115^ ^]^ Unlike battery‐less devices, battery‐free devices do not incorporate any integrated power storage but rely on continuous wireless power transfer (e.g., magnetic resonant coupling) to operate in real time. As shown in Figure 9b, Noh et al. reported a miniaturized, battery‐free, wireless optofluidic system based on wireless RF power transmission.^[^ ^9^ ^]^ The device achieves power transfer exceeding 2 W while implanted in a mouse brain, sufficient for most bioelectronic applications.

## 4 Challenges in Clinical Translation

Despite extensive research and technical advancement achieved in the field of closed‐loop biomedical systems, both self‐sustained and externally triggered systems are faced with some challenges toward further clinical translations. In this section, major hurdles in clinical translation, including biocompatibility concerns, long‐term stability and system lifetime, and regulatory complications, are evaluated and discussed.

In state‐of‐the‐art self‐sustained closed‐loop biomedical systems, biocompatibility and toxicity from the drug cargo are one of the main drawbacks. In the operation of GOx‐based glucose‐responsive systems, hydrogen peroxide is produced as a side product, which may cause detrimental effects and cytotoxicity at high concentrations.^[^ ^1^ ^]^ Meanwhile, immune responses and toxicity of GOx, Con A, and PBA have been reported but are not yet fully understood.^[^ ^116^, ^117^ ^]^ For successful clinical translation of these self‐sustained closed‐loop systems, extensive investigations of biocompatibility and safety are required. Regarding externally triggered closed‐loop systems, issues related to biocompatibility and toxicity vary significantly depending on the specific choice of sensors, active treatment, data, and power transmission approaches. As individual components in an externally triggered system operate independently, it is feasible to alternate between options and circumvent biocompatibility issues. Cases of externally triggered systems have been reported where biocompatibility has been evaluated and validated.^[^ ^7^, ^108^ ^]^

In terms of stability and lifespan of closed‐loop systems, the externally triggered system generally exhibits better performance compared to self‐sustained systems. The majority of self‐sustained glucose‐responsive systems rely on molecules and enzymes as a responsive mechanism. As a result, these systems are inherently vulnerable to degradation and more sensitive to changes in the body's environment.^[^ ^26^ ^]^ On the other hand, externally triggered systems achieve self‐regulated treatment through external controls, which allows for more stable operation and an elongated lifespan.

As novel biomedical treatment approaches, both types of closed‐loop systems may encounter rigorous regulatory investigations and delay their adoption into clinical applications. In the case of self‐sustained systems, the complexity of the closed‐loop mechanism implies further complications in regulatory approval processes. Each aspect of the systems, from drug and cargo safety to the reliable operation of self‐regulation, will require in‐depth investigations and pose difficulties to clinical translation. In externally triggered systems, sensors, active treatments, and data/power transmission are individually developed and then integrated as a closed‐loop system. Regulation protocols are more developed and mature for individual components, especially in bioelectronic devices. Therefore, less difficulty in staying compliant with regulatory requirements is expected in the research and development process.

## 5 Conclusion and Outlook

Closed‐loop systems have emerged as a transformative approach in precision therapy, integrating diverse active treatment strategies to deliver personalized medicine. This review has highlighted the significant progress in self‐sustained and externally triggered systems, two main types of closed‐loop systems, emphasizing their mechanisms, material formulation, device fabrication, and potential applications. Innovations such as glucose‐responsive drug delivery systems and bioelectronics‐based devices have demonstrated how advanced biomaterials and device designs enable precise control over therapeutic interventions, paving the way for more efficient and personalized healthcare. For self‐sustained closed‐loop systems, the development of bio‐responsive materials is critical. These materials allow for controlled drug release through structural transformations, such as shrinking, swelling, or degradation, in response to internal bio‐signals associated with disease states. Externally triggered systems, on the other hand, rely on advanced bioelectronics to dynamically adjust therapy based on real‐time feedback. These systems incorporate sophisticated components such as sensors, processors, and actuators to monitor, analyze, and respond to physiological changes. They offer enhanced precision, enabling therapies tailored to individual needs. These intelligent devices have shown potential to improve therapeutic efficiency and reduce adverse effects, with applications spanning diabetes management, auto‐anticoagulation regulation, optogenetic stimulation for neuromuscular control, wound treatment, and beyond. The major advantages and disadvantages of self‐sustained and externally triggered closed‐loop systems are summarised in comparison in **Table** **1**.

**Table 1:** Comparative table of self‐sustained and externally triggered closed systems.^[^ ^1^, ^17^, ^26^, ^27^, ^82^, ^83^, ^93^, ^118^ ^]^

System type	Real‐time regulation	Personalised treatment	Response time	Burden of Care	Long‐term stability and lifespan
Self‐sustained systems	Yes	Yes	Medium	Minimal	Limited
Externally‐triggered systems	Yes	Yes	Fast	Reduced compared to conventional treatments	Good
System type	Invasiveness	Treatment options	Miniaturization	Biocompatibility	Clinical feasibility
Self‐sustained systems	Non‐invasive	limited	Readily miniaturized as drug‐based systems	May induce adverse immune responses	Challenges in clinical translation
Externally‐triggered systems	Minimally invasive	Compatible with various treatments	Challenge in miniaturization of devices	Good biocompatibility	At the early stage of clinical trials

Despite remarkable advancements over the past decade, several challenges remain. Achieving long‐term stability and sustained biocompatibility is essential to support continuous monitoring, diagnosis, and treatment. New material formulations must be carefully assessed to eliminate toxic byproducts and ensure patient safety. In addition, sensor reliability and the ability to accurately define normal and abnormal physiological conditions across diverse populations are critical hurdles that must be addressed. Furthermore, integrating and miniaturizing bioelectronics‐based systems for seamless functionality within the body remain key technical barriers.

Looking ahead, several emerging technologies promise to further revolutionize closed‐loop therapies. Technologies such as two‐photon polymerization (2PP) 3D printing now enable the creation of high‐resolution structures at sub‐micron scales, offering flexibility for a wide variety of 3D configurations.^[^ ^119^ ^]^ The material library for 2PP systems has expanded from purely biocompatible materials to multifunctional options, facilitating more versatile and complex applications.^[^ ^120^ ^]^ Additionally, soft robotics is emerging as a transformative technique, converting conventionally passive bioelectronic devices into active systems capable of adapting to intricate biological surfaces and dynamic environments.^[^ ^121^ ^]^ At the same time, the integration of artificial intelligence into closed‐loop systems holds immense potential to enhance adaptability and decision‐making.^[^ ^122^ ^]^ Artificial intelligence can be integrated directly into closed‐loop systems for predictive therapies, which enables the system to anticipate physiological perturbations and adjust treatment accordingly.

Finally, translating these innovations from bench to bedside will require more than just scientific breakthroughs, which demands a steadfast commitment to patient‐centric design. Usability, affordability, accessibility, and seamless integration into everyday life are vital to drive adoption and ensure patient compliance. Devices must be intuitive to operate, comfortable to wear or implant, and adaptable to diverse demographics and clinical settings. Equally important are cost‐effective manufacturing and distribution strategies that broaden access, especially in resource‐limited environments. By prioritizing these human‐focused considerations alongside technological advances, closed‐loop systems can move from promising prototypes to impactful, real‐world therapies.

## Conflict of Interest

The authors declare no conflict of interest.
